# Ultra-rapid glutathionylation of chymotrypsinogen in its molten globule-like conformation: A comparison to archaeal proteins

**DOI:** 10.1038/s41598-020-65696-5

**Published:** 2020-06-02

**Authors:** Alessio Bocedi, Giorgia Gambardella, Giada Cattani, Simonetta Bartolucci, Danila Limauro, Emilia Pedone, Federica Iavarone, Massimo Castagnola, Giorgio Ricci

**Affiliations:** 1 Dipartimento di Scienze e Tecnologie Chimiche, Università di Roma “Tor Vergata”, Rome, Italy; 2 Dipartimento di Biologia, Università di Napoli Federico II, Naples, Italy; 3 Istituto di Biostrutture e Bioimmagini, C.N.R., Naples, Italy; 4 Istituto di Biochimica e Biochimica Clinica, Università Cattolica del Sacro Cuore, Rome, Italy; 5 IRCCS Fondazione Policlinico Universitario Agostino Gemelli, Rome, Italy; 6 Laboratorio di Proteomica, Centro Europeo di Ricerca sul Cervello, IRCCS Santa Lucia, Rome, Italy

**Keywords:** Protein folding, Chemical biology

## Abstract

Chymotrypsinogen, when reduced and taken to its molten globule-like conformation, displays a single cysteine with an unusual kinetic propensity toward oxidized glutathione (GSSG) and other organic thiol reagents. A single residue, identified by mass spectrometry like Cys1, reacts with GSSG about 1400 times faster than an unperturbed protein cysteine. A reversible protein-GSSG complex and a low p*K*_a_ (8.1 ± 0.1) make possible such astonishing kinetic property which is absent toward other natural disulfides like cystine, homocystine and cystamine. An evident hyper-reactivity toward 5,5′-dithiobis-(2-nitrobenzoic acid) (DTNB) and 1-chloro-2,4-dinitrobenzene (CDNB) was also found for this specific residue. The extraordinary reactivity toward GSSG is absent in two proteins of the thermophilic archaeon *Sulfolobus solfataricus*, an organism lacking glutathione: the Protein Disulphide Oxidoreductase (*Ss*PDO) and the Bacterioferritin Comigratory Protein 1 (Bcp1) that displays Cys residues with an even lower p*K*_a_ value (7.5 ± 0.1) compared to chymotrypsinogen. This study, which also uses single mutants in Cys residues for Bcp1, proposes that this hyper-reactivity of a single cysteine, similar to that found in serum albumin, lysozyme, ribonuclease, may have relevance to drive the “incipit” of the oxidative folding of proteins from organisms where the glutathione/oxidized glutathione (GSH/GSSG) system is present.

## Introduction

A few proteins with structural disulfide bonds in the native status, like albumin, lysozyme and ribonuclease, when reduced and taken to a molten globule-like conformation, display a never observed ultra-rapid interaction of a single (or a few) cysteines with oxidized glutathione (GSSG)^1–3^. This phenomenon does not occur with other natural disulfides and is promoted by the synergic combination of a specific transient protein-GSSG complex and a low p*K*_a_ value of the hyper-reactive cysteines. The astonishing entity of this phenomenon, quantified in terms of thousand times increased reactivity toward GSSG, leads to an almost instantaneous glutathionylation of a single cysteine. This reaction is intriguing as GSSG is present at high concentrations in the endoplasmic reticulum where the oxidative folding occurs^4,5^ and thus a specific glutathionylation may represent the “incipit” of this process as it may happen at great speed without the assistance of some enzyme catalysis^3^. We remember, in fact, that a few details of this fundamental mechanism for many proteins are still to be clarified. For many years, GSSG was thought to be the prime source of oxidative equivalents for the protein disulfide formation, but starting from twenty years ago the endoplasmic reticulum oxidase (Ero1) and the protein disulfide isomerase (PDI) were indicated as the true actors in this oxidative mechanism^6–8^. Now, we do not want to enter the complex debate about the ultimate electron acceptors for the disulfide formation, but only to signal an unknown and very strange property that may be present in other proteins. In fact, this study reveals that besides lysozyme, albumin and ribonuclease, also chymotrypsinogen A (ChTg) (ten cysteines forming five disulfides) shows a single hyper-reactive cysteine toward GSSG. The observation that this phenomenon is absent in ancestral disulfide archaeal enzymes which are produced in organism lacking GSSG and glutathione (GSH)^9^ like the *Sulfolobus solfataricus* Bacterioferritin Comigratory Protein 1 (Bcp1)^10,11^ and Protein Disulfide Oxidoreductase (*Ss*PDO)^12,13^ will give some possible interpretation for the presence or absence of this property in proteins.

**Figure 1:**
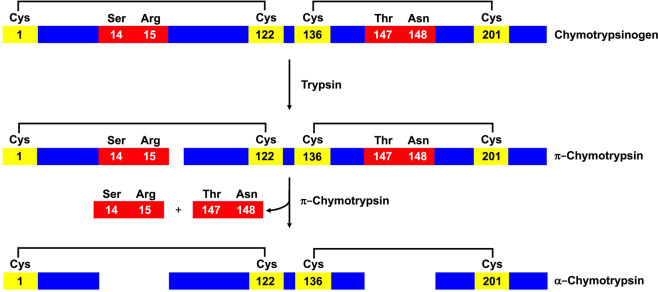
Representative scheme of α-chymotrypsin activation. Residues that undergo hydrolysis are represented in red, cysteines are shown in yellow and the black lines represent the disulfide bridges.

Chymotrypsin is an endopeptidase with five disulfide bridges that hydrolyzes peptides involving aromatic amino acids like tyrosine, phenylalanine and tryptophan. This enzyme is synthetized like inactive ChTg in the pancreas with an 18-amino acid signal peptide, 15-amino acid activation peptide and a 230-amino acid active molecule. ChTg displays also five disulfides. The activation to α-chymotrypsin is achieved by the hydrolytic breakdown between Arg15 and Ile16. The small fragment 1–15 remains bound to the active molecule through a Cys1-Cys122 disulfide bridge^14,15^ (Fig. 1). Thus, this disulfide must be formed early before the proteolytic event.

Only a few studies have been performed about the oxidative folding of this pro-enzyme also due to a strong propensity of the reduced enzyme to form irreversible and insoluble aggregates. One interesting report detailed the renaturation pathway of reduced ChTg (rChTg) in 1 M guanidine in the presence of a GSH/GSSG mixture, observing that the enzyme regained the correct disulfide pairing in a few hours^16^. No studies have been done about the intrinsic reactivity of specific cysteines toward GSSG and thus the present report is finalized to fill the gap that could reveal the “incipit” of the oxidative mechanism that is unknown until today.

## Results

### Reaction of rChTg with DTNB and other thiol reagents

ChTg was reduced with dithiothreitol (DTT) in 8 M urea at pH 8.0 as described under Materials and Methods. After removal of DTT by a G-25 Sephadex column, the titration of the reduced cysteines was done using 5,5′-dithiobis-(2-nitrobenzoic acid) (DTNB). When the assay was performed at pH 8.0 in 8 M urea, 9.8 ± 0.3 -SH/mole of enzyme were titrated and a similar value (9.6 ± 0.4) was obtained by reacting DTNB with rChTg in 0.2 M urea at pH 5.0, a condition that leads the enzyme to form linear soluble aggregates^17^. The presence of soluble oligomers, similar to those observed for the partially reduced ChTg^17^ was suggested by CD spectroscopic evidence (below reported) and by their permanence in solution after centrifugation.

**Figure 2:**
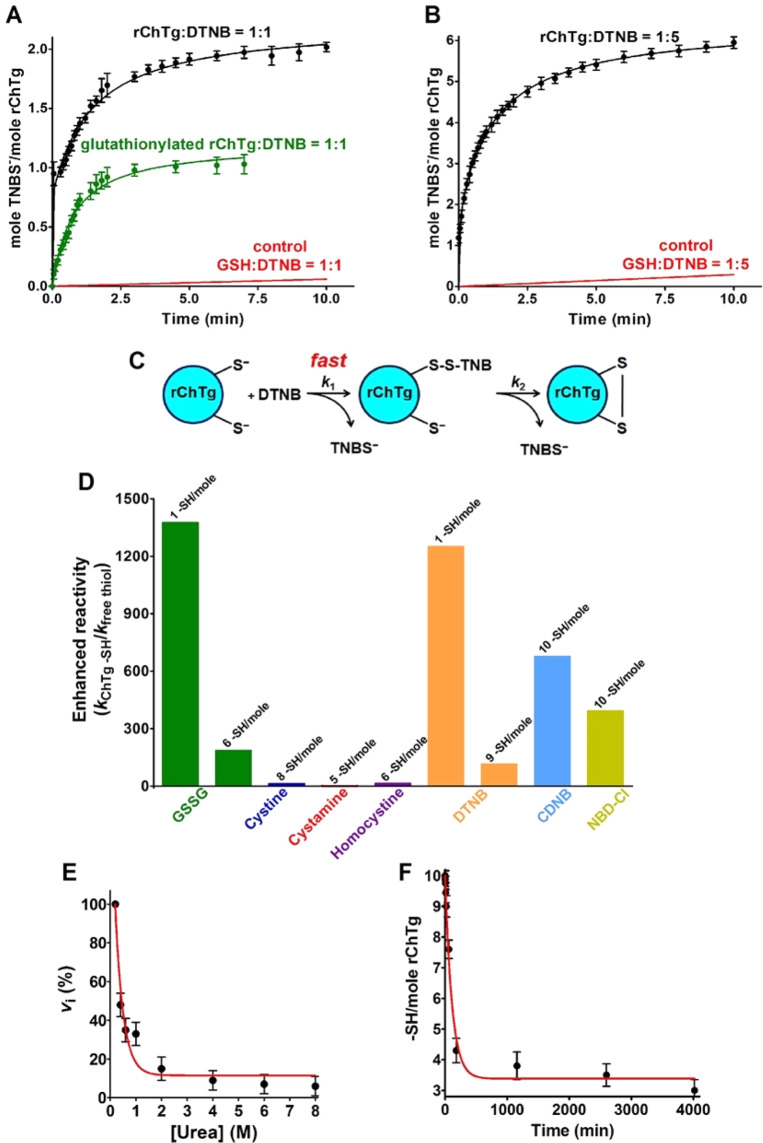
Reactivity of rChTg cysteines. (**A**) TNBS^-^ release after reaction of rChTg (0.58 µM, 5.8 µM protein -SH) with substoichiometric DTNB (0.58 µM) in 50 mM acetate buffer at pH 5.0, 0.2 M urea 25 °C (black line). The same rection as above but after 20 min incubation of 0.58 µM of rChTg with 1 mM GSSG (green line). Reaction of GSH (5.8 µM) with DTNB (0.58 µM) in the same conditions (red line). (**B**) TNBS^-^ release after reaction of rChTg (0.58 µM, 5.8 µM protein -SH) with substoichiometric DTNB (2.9 µM) in 50 mM acetate buffer at pH 5.0, 0.2 M urea 25 °C. Reaction of GSH (5.8 µM) with DTNB (2.9 µM) in the same conditions (red line). (**C**) Schematic representation of the reaction of rChTg with stoichiometric DTNB. (**D**) “Enhanced reactivity” of rChTg toward disulfides and thiol reagents *i.e*. second order kinetic constants of rChTg (*k*_ChTg -SH_) normalized to the constant calculated for an unperturbed protein cysteine for GSSG or normalized to the constants for free GSH for all other reagents (*k*_free thiol_) (see Table 1). The number of protein cysteines per mole with a given reactivity is indicated on the top of each column. (**E**) Reactivity of cysteines in rChTg (0.6 µM) toward DTNB (47.5 μM) at variable urea concentrations (pH 5.0) (25 °C) (circles, red line). Rate of reaction of 10 µM free cysteine (or GSH) with 50 µM DTNB was not inhibited by 8 M urea (not shown). The error bars represent the S.D. from three independent experiments. (**F**) Disappearence of rChTg cysteines (5 µM, 50 µM protein -SH) during the reaction with 1 mM GSSG at pH 5.0, 0.2 M urea (25 °C). The error bars represent the S.D. from three independent experiments.

The reaction performed using sub-stoichiometric DTNB concentrations gave interesting information. In fact, using a DTNB: rChTg 1:1 (DTNB:protein cysteines 1:10), two 5-thio-2-nitrobenzoate (TNBS^-^) ions were released (Fig. 2A) with a typical biphasic kinetics: after a first very fast production of a mixed disulfide rChTg-SS-TNB, a second protein cysteine slowly forms an internal protein disulfide releasing a second TNBS^**-**^ ion. This phenomenon does not occur for other protein cysteines. In fact, when incubated in a ratio DTNB: rChTg 5:1, (DTNB:protein cysteines 5:10) the release of only 6 TNBS^-^ ions was observed (Fig. 2B). This behaviour likely suggests that only a single cysteine reacts with DTNB when rChTg is incubated in a stoichiometric amount with DTNB and that this residue must display hyper-reactivity toward DTNB compared to all other cysteines (Fig. 2C). As it will be demonstrated below, the actual presence of a single hyper-reactive cysteine will be definitively proved using mass spectrometry experiments. The other 4 protein cysteines remain tied to a TNB residue like mixed disulfides without evolving toward a protein disulfide. By examining the kinetics of the reaction with an excess of DTNB, it is evident that the single hyper-reactive cysteine, as well as the other nine cysteines, react with extraordinary velocity. Evaluation of the corresponding kinetic constants (Table 1) indicates a 1250 and 115 fold higher reactivity, for one and the nine remaining cysteines, respectively (Fig. 2D). This hyper-reactivity is likely made possible by some useful property of a partially folded structure because in 8 M urea all cysteines display a very limited reactivity (about 5%) (Fig. 2E).

**Table 1:** Reactivity of cysteines of fully reduced ChTg toward thiol reagents, pH 5.0.

Reagents	Free Cys	Free GSH	Reduced ChTg			
	*k* (M^−1^ s^−1^)	*k* (M^−1^ s^−1^)	*k* (M^−1^ s^−1^) (-SH/mol ChTg)	E.R.	*k* (M^−1^ s^−1^) (-SH/mol ChTg)	E.R.
GSSG	0.0028^a^ 0.0008^b^	—	1.1 ± 0.1 (1)	393 1375	0.15 ± 0.03 (6)	54 187
Cystine	—	0.048	0.56 ± 0.02 (8)	12	—	—
Cystamine	—	0.22	0.05 ± 0.01 (5)	0.23	—	—
Homocystine	—	0.0024	0.037 ± 0.003 (6)	15	—	—
DTNB	—	20	25000 ± 1000 (1)	1250	2300 ± 100 (9)	115
DTNB (8 M urea)	—	20	300 ± 20 (4) 5 ± 1 (6)	15 0.25	—	—
CDNB	—	0.00028	0.19 ± 0.01 (10)	678	—	—
CDNB (8 M urea)	—	0.00028	0.0003	1	—	—
NBD-Cl	—	0.032	12.6 ± 0.5 (10)	394	—	—

^a^Experimental value for free cys (p*K*_a_ = 8.55);

^b^Theoretical value for unperturbed protein cysteine (p*K*_a_ = 9.1);

E.R., Enhanced reactivity;

Data are Mean ± S.D.

We did not perform experiments in 1 M in guanidine as previously made^16^, but we preferred to operate at pH 5.0 and 0.2 M urea where the enzyme forms semi-flexible amyloid polymers^17^ which remain in solution and do not perturb spectroscopic and kinetic analysis.

A relevant hyper-reactivity of all cysteines was also observed toward 1-chloro-2,4-dinitrobenzene (CDNB) and 4-chloro-7-nitrobenzofurazan (NBD-Cl), two thiol reagents with hydrophobic properties. Both reagents were able to interact with all 10 protein cysteines. In Table 1 and Fig. 2D we reported an average hyper-reactivity for all ten cysteines.

### Reaction of rChTg with GSSG and other natural disulfides

GSSG is particularly abundant in the endoplasmic reticulum (from 0.4 mM to 2 mM)^4,5^ and thus its possible interaction with rChTg may really occur in the cell during the nascent phase.

As shown in Fig. 2F and Table 1, GSSG reacts with 7 of the 10 protein cysteines at pH 5.0. The average kinetic constant calculated for all cysteines was 0.15 M^−1^ s^−1^ indicating a 190 times enhanced reactivity when compared to a theoretical unperturbed protein cysteine. Mass spectrometry will demonstrate that after 20 min of incubation only a single cysteine was modified, so from the initial rate of the kinetic trend reported in Fig. 2D and Table 1, a 1375 times enhanced reactivity was reasonably calculated for this residue. This phenomenon is specific for GSSG because other natural disulfides like cystine, cystamine and homocystine show no or very scarce reactivity toward rChTg (Table 1).

A second convincing evidence was that GSSG, during 20 min, interacts mainly with the same cysteine which was found hyper-reactive toward DTNB. After this reaction with GSSG, there is the absence of the ultra-rapid cysteine that, without GSSG treatment, reacts with DTNB within the instrumental dead-time (Fig. 2A). The three unreactive cysteines toward GSSG are probably surrounded by hydrophobic residues favoring a positive hydrophobic interaction with DTNB, CDNB and NBD-Cl but not with GSSG.

### Cys1 is the most hyper-reactive residue toward GSSG in rChTg

At first, a high-resolution nano-HPLC-ESI-MS analysis was carried out on the intact ChTg. The deconvolution of the ESI spectra, carried out by Xtract software (Thermofisher) on the almost unique HPLC peak, provided a [M + H]^1+^ = 25640.61 *m/z*, in optimal agreement with the theoretical value (25640.69 *m/z*) of the non-processed protein (Swiss Prot Code: P00766).

**Figure 3:**
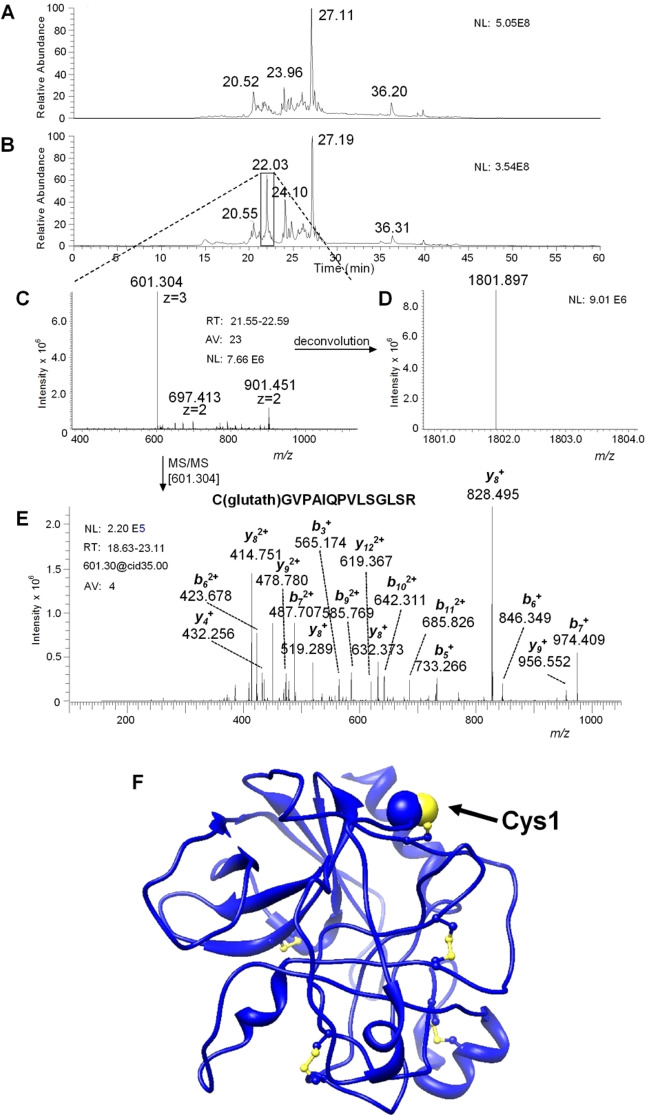
MS and MS/MS analysis. (**A**) Total ion current (TIC) nano-HPLC-ESI-MS profile of the tryptic digest of reduced chymotrypsinogen A and treated with bromopyruvic acid. (**B**) TIC nano-HPLC-ESI-MS profile of the tryptic digest of reduced chymotrypsinogen A and treated with glutathione before the reaction with bromopyruvic acid. The comparison of the two profiles evidenced a new peak with an elution time of 22.03 min in the tryptic digest. (**C**) The peak was related to a peptide with a [M + 3 H]^3+^ = 601.304 m/z (and a [M + 2 H]^2+^ = 901.451 m/z). (**D**) The deconvolution provided the monoisotopic [M + H]^1+^ = 1801.897 m/z of the peptide. (**E**) The collision induced dissociation CID MS/MS carried out on the ion [M + 3 H]^3+^ = 601.304 m/z, elaborated by manual inspection and by the Proteome Discover software, was in perfect agreement with the theoretical fragmentation of the tryptic peptide C(Gluthat)GVPAIQPVLSGLSR. This peptide corresponds to amino acid residues 1–15 of chymotrypsinogen A bovine with a glutathione residue linked to Cys1 by a disulfide bridge. (**F**) Three-dimensional structure of native ChTg from bovine pancreas is represented in blue ribbons; cysteines are in ball-and-stick, the Cys1 is displayed by blue and yellow spheres.

To verify whether this hyper-reactivity is maximally localized in a single residue, as indicated by the reaction with DTNB and GSSG or, *vice versa*, in multiple cysteines, rChTg was reacted with 1 mM GSSG at pH 5.0. After 20 min (one Cys/mole reacted), the unreacted cysteines were instantaneously alkylated with bromopyruvate. The modified protein was proteolyzed with trypsin and the resulting peptides separated and analyzed by mass spectrometry as described in the Materials and Methods. The comparison between the HPLC total ion current (TIC) profiles of the trypsin digest of the rChTg treated with only bromopyruvate with that one of rChTg reacted with GSSG before the treatment with bromopyruvate allowed the detection of a peak only present in the tryptic digest of rChTg treated with GSSG (Elution time 22.03 min) (Fig. 3A,B). The principal peak ions were the [M + 3 H]^3+^ = 601.30 *m/z* ion and the [M + 2 H]^2+^ = 901.44 *m/z* ion (Fig. 3C), pertaining to a tryptic peptide with [M + H]^1+^ = 1801.90 *m/z* (Fig. 3D). The automatic interpretation of the MS/MS spectrum (Fig. 3E) of the [M + 3 H]^3+^ = 601.30 *m/z* ion carried out by Proteome Discoverer 1.4 software (Thermofisher) established that the peptide corresponded to the tryptic fragment 1–15 of ChTg with a glutathione residue linked to Cys1 by a disulfide bridge (Fig. 3E,F). The manual inspection of the MS/MS spectrum of the [M + 2 H]^2+^ = 901.44 *m/z* ion confirmed this interpretation, as well as the manual inspection of the MS/MS spectrum of the ion at [M + 3 H]^3+^ = 601.30 *m/z*.

### pK_a_ determination of rChTg cysteines

**Figure 4:**
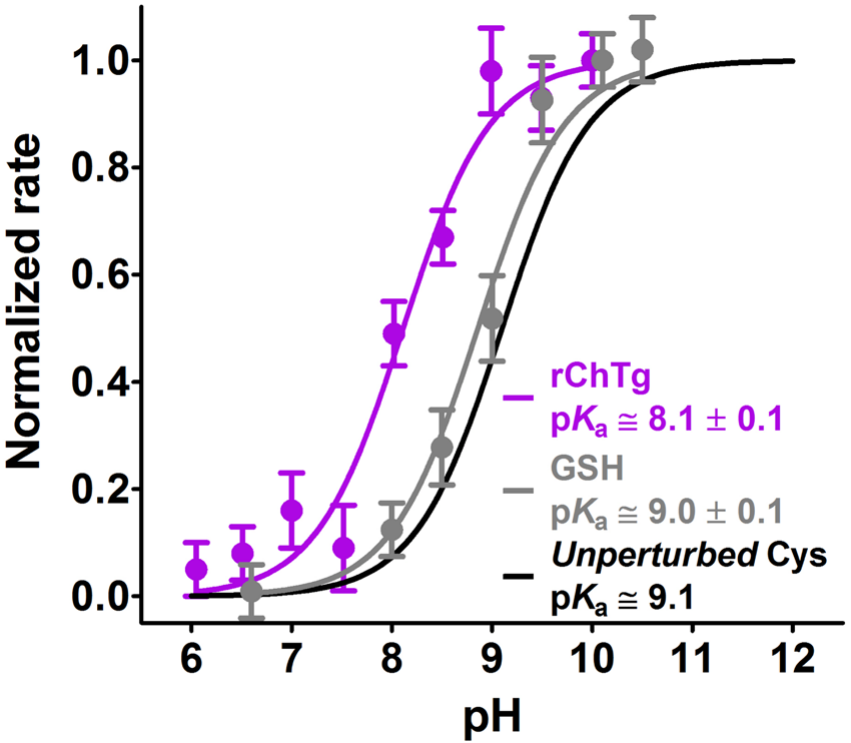
Average p*K*_a_ determination of ChTg. rChTg (0.6 µM) was reacted with CDNB (1 mM) at variable pH values (purple line). Average p*K*_a_ = 8.1 ± 0.1 of the ten reactive cysteines in rChTg. As a control experiment GSH (100 µM) was reacted with CDNB (1 mM) at variable pH values (gray line). The error bars represent the S.D. from three independent experiments. The theoretical curve (black line) of an unperturbed protein cysteine (p*K*_a_ = 9.1) is also reported.

The p*K*_a_ determination was performed using CDNB as thiol reagent. DTNB cannot be used as its reaction at neutral and alkaline pH is too fast to be followed without a stopped-flow apparatus. Kinetic data obtained at 340 nm (where the cysteine-DNB adduct absorbs) were subtracted by the slight absorbance perturbation due to aggregation at neutral and alkaline pH values. From the variation of the observed velocity on pH (Fig. 4), an average p*K*_a_ of 8.1 ± 0.1 was calculated which represents a lowered p*K*_a_ of about 1 unit when compared to the one of a theoretical unperturbed protein cysteine (9.1)^18^, or of GSH (9.0 ± 0.1). This variation can lead to only a ten fold increased reactivity as much as (see Supplementary material in ref. ^2^), so other factors must be present to produce hundreds and even thousands of times higher reactivity toward GSSG and other thiol reagents.

### Evidence for a reversible rChTg-GSSG complex

**Figure 5:**
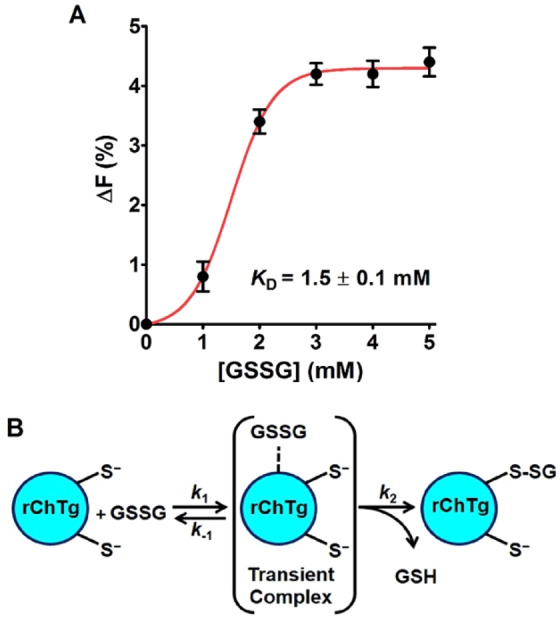
Transient complex formation with GSSG. (**A**) Quenching of the intrinsic fluorescence of rChTg (0.5 µM) after addition of GSSG (pH 5.0, 25 °C) subtracted from the fluorescence of NATA with GSSG. The error bars represent the S.D. from three independent experiments. (**B**) Representative reaction scheme of rChTg with GSSG. The glutathionylation occurs at the most reactive cysteine.

Due to the facile aggregation of the reduced enzyme at neutral pH values into insoluble polymers^19^, all fluorescence experiments were performed at pH 5.0 observing the quenching of the intrinsic fluorescence after suitable addition of GSSG. The fluorescence perturbation was monitored at 295 nm after a few seconds from any addition (Fig. 5A), to avoid possible quenching due to the glutathionylation of Cys1, a reaction which is negligible within 5–10 sec after addition of GSSG. The fluorescence perturbation at increasing GSSG concentrations follows a sigmoidal trend with an apparent *K*_D_ of 1.5 mM. The saturation behavior represents a strong indication that GSSG forms a reversible rChTg-GSSG complex before the glutathionylation of Cys1 (Fig. 5B). We underline that this *K*_D_ is obtained at pH 5.0, a value far from the physiological one and then these non-physiological conditions may have a negative influence on the affinity for GSSG which may be higher at pH 7.0.

### Circular dichroism spectra of ChTg

**Figure 6:**
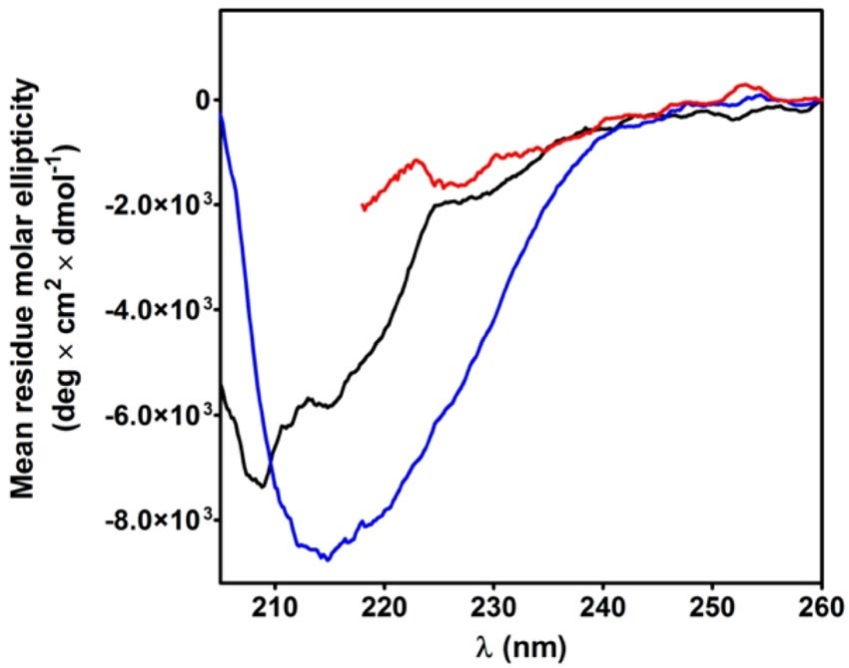
Circular dichroism spectra of ChTg and rChTg. CD spectra of native ChTg (1.3 µM) (black line); rChTg (1.3 µM) in 0.2 M urea (blue line) and rChTg (1.3 µM) in 8 M urea (red line). CD spectra were recorded at pH 5.0, 25 °C. The spectrum of rChTg in 8 M urea cannot be extended below 218 nm due to the interference of 8 M urea.

The circular dichroism (CD) spectra of the native ChTg, and rChTg in 0.2 M urea or 8 M urea are reported in Fig. 6. Surprisingly, the reduced conformation shows an apparent higher structuration when compared to the native enzyme. This spectral evidence, which confirms what reported in previous studies^17^, is due to a slightly increase of β-sheet signal. In fact, the analysis of the CD spectra, made as described under Materials and Methods section, showed an increased value of β-sheet (39%) in the reduced protein compared to the 25% of the native form while the α-helix slightly decreases from 12% to 10% in the reduced form.

### Two proteins from the thermoacidophilic archaeon *Sulfolobus solfataricus*

One possible role of the hyper-reactivity toward GSSG is to prevent or limit the formation of non-natural disulfides and to speed the disulfide formation. If this hypothesis is correct, reduced proteins in a molten globule-like conformation that are produced in organisms lacking glutathione, would not display the hyper-reactivity toward GSSG like found in rChTg.

Bacterioferritin Comigratory Protein 1 (Bcp1) (1 disulfide) and Protein Disulphide Oxidoreductase (*Ss*PDO) (3 disulfides) from *S. solfataricus* are presented as useful models for this purpose because in this thermoacidophilus archaeon glutathione is absent^9,12^. In addition, in Bcp1 the possibility of incorrect disulfides is null, showing only two cysteines.

### Bcp1 reactivity

**Figure 7:**
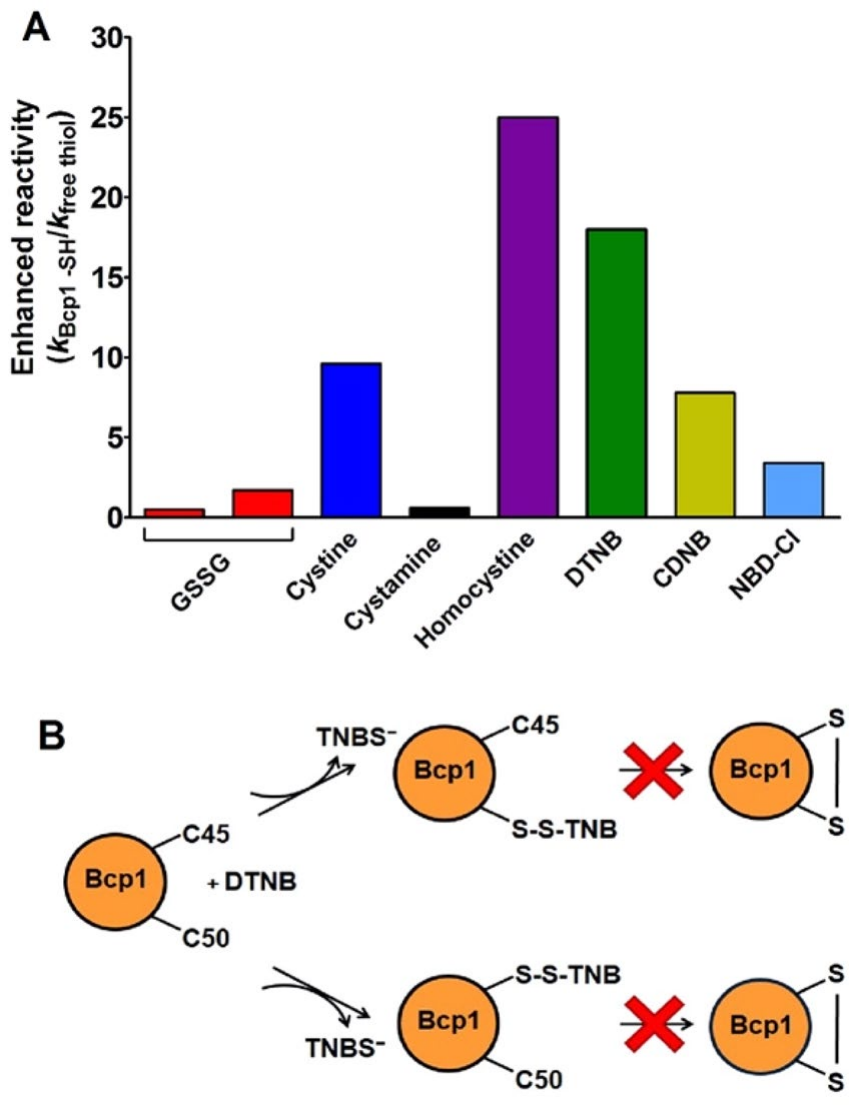
Reactivity of rBcp1 cysteines. (**A**) “Enhanced reactivity” of rBcp1 toward disulfides and thiol reagents *i.e*. second order kinetic constants of rBcp1 (*k*_Bcp1 -SH_) normalized to the constant calculated for an unperturbed protein cysteine for GSSG or normalized to the constants for free GSH for all other reagents (*k*_free thiol_) (see Table 2). (**B**) Schematic representation of the reaction of native reduced Bcp1 with DTNB, the formation of the intramolecular disulfide does not proceed in the molten globule-like state (see Table 2).

Expression, purification and reduction of the native Bcp1 were done as reported under Materials and Methods. Surprisingly, both its cysteines can be titrated with DTNB only in 10 M urea. Conversely, in 0.2 M urea only one cysteine reacts with DTNB as well as with all other tested reagents. As expected, no hyper-reactivity was recovered for GSSG and other natural disulfides except for a very slight over-reactivity for homocystine (Table 2 and Fig. 7A).

**Table 2:** Reactivity of cysteines of native Bcp1 and its mutants toward thiol reagents, pH 7.4.

Reagents	Free Cys	Free GSH	Reduced Bcp1		Reduced C45S		Reduced C50S	
	*k* (M^−1^ s^−1^)	*k* (M^−1^ s^−1^)	*k* (M^−1^ s^−1^) (-SH/mole Bcp1)	E.R.	*k* (M^−1^ s^−1^) (-SH/mole Bcp1)	E.R.	*k* (M^−1^ s^−1^) (-SH/mole Bcp1)	E.R.
GSSG	0.7^a^ (0.2)^b^	—	0.35 ± 0.02 (1)	0.5 1.7	—	—	—	—
Cystine	—	12	115 ± 10 (1)	9.6	4.2 ± 0.4 (0.6)	0.35	3.5 ± 0.2 (0.65)	0.29
Cystamine	—	55	33 ± 3 (1)	0.6	2.1 ± 0.2 (0.6)	0.04	3.5 ± 0.1 (0.65)	0.06
Homocystine	—	0.6	15 ± 1 (1)	25	3.8 ± 0.2 (0.6)	6.3	0.5 ± 0.1 (0.65)	0.8
DTNB								
(pH 5.0)	—	20	360 ± 20 (1)	18	—	—	—	—
DTNB (pH 7.4)	—	5000	—	—	1400 ± 200 (0.6)	0.28	—	—
DTNB (pH 6.0)	—	150	—	—	—	—	925 ± 50 (0.65)	6.2
CDNB	—	0.07	0.55 ± 0.03 (1)	7.8	0.18 ± 0.02 (0.6)	2.6	0.39 ± 0.02 (0.65)	5.6
NBD-Cl	—	8	27 ± 3 (1)	3.4	3.4 ± 0.2 (0.6)	0.4	4.9 ± 0.2 (0.65)	0.6

^a^Experimental value for free cys (p*K*_a_ = 8.55);

^b^Theoretical value for unperturbed protein cysteine (p*K*_a_ = 9.1);

E.R., Enhanced reactivity;

Data are Mean ± S.D.

**Figure 8:**
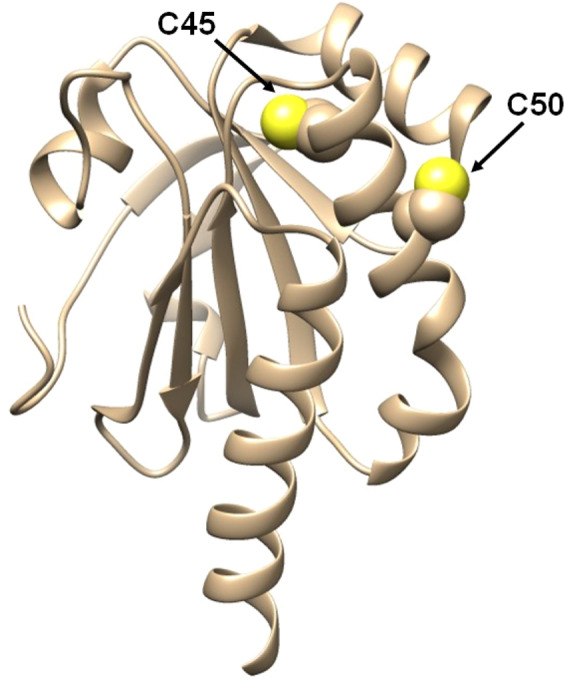
Three-dimensional structure of native Bcp1 from *Sulfolobus solfataricus* is represented in light brown ribbons. The sulfur atoms of the two cysteines are shown as yellow spheres.

However, to deeply investigate the role of the single cysteine reactivity of the reduced Bcp1 (rBcp1) we purified two protein mutants. The first one was the C45S in which the peroxidatic cysteine, involved in the catalytic mechanism, was mutagenized; while C50S showed the substitution of the resolving cysteine^11^ (Fig. 8).

In both mutants, no cysteines were titratable by DTNB. The denaturation performed under reducing conditions for both mutants allowed us to titrate one cysteine per mutant in 10 M urea. Surprisingly in 0.2 M urea, both the reduced mutants (rC45S and rC50S) also display about one reactive cysteine, while one mutant was expected unreactive toward DTNB given that, under similar conditions, only one cysteine is titratable in the native enzyme. This paradox may be easily explained assuming that both these cysteines equally react with DTNB but, after one residue has reacted, the other one becomes unavailable to this reagent, possibly for steric reasons (Fig. 7B).

Both mutants show also an absence of hyper-reactivity toward disulfides very similar to that found in the native enzyme (Table 2).

### p*K*_a_ determination of rBcp1

**Figure 9:**
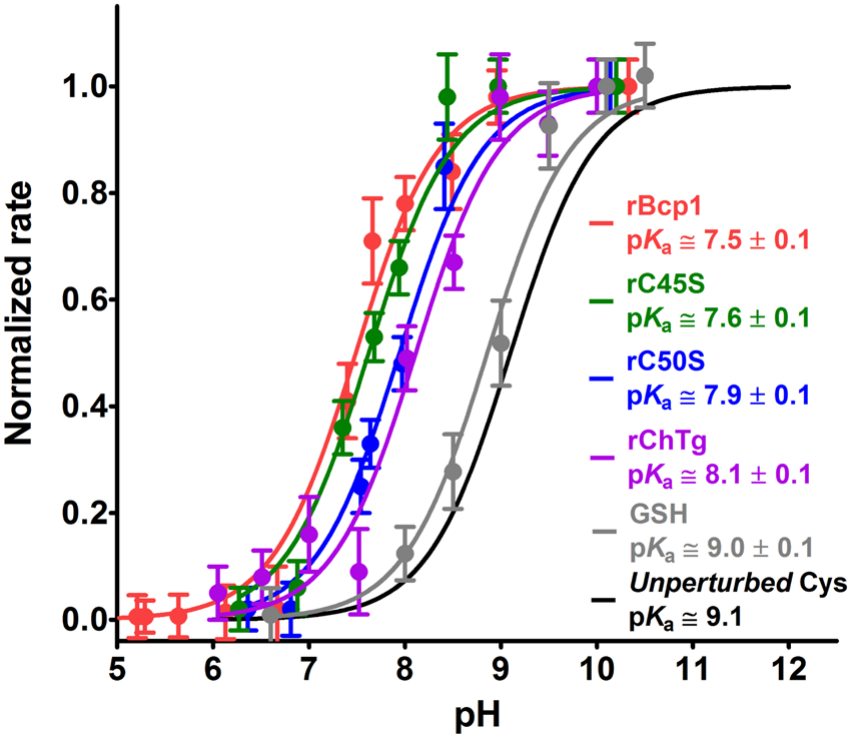
Average p*K*_a_ determination of Bcp1. rBcp1 (1.25 μM) (red line), rC45S (2.6 μM) (green line) and rC50S (2.6 μM) (blue line) were reacted with DTNB (20 μM) at variable pH values (25 °C). Average p*K*_a_ values of the reactive cysteine are reported within the graph according with curves colours. As a control experiment, GSH (100 µM) was reacted with CDNB (1 mM) at variable pH values (gray line). The error bars represent the S.D. from three independent experiments. The reference curve (from Fig. 4) of rChTg reacted with CDNB at variable pH values (purple line) is displayed. The theoretical curve (black line) of an unperturbed protein cysteine (p*K*_a_ = 9.1) is also reported.

The p*K*_a_ value of the apparent single residue able to react with DTNB in the native enzyme was determined on the basis of the rate trend at different pH values. As shown in Fig. 9 a value of 7.5 was estimated. This corresponds to about 1.6 lower unit compared to the one of an unperturbed protein cysteine. Arg112 and Arg53 are the only positively charged residues that may concur to lower the p*K*_a_ of both Cys45 and Cys50. Similar experiments performed on the C45S and C50S give p*K*_a_ value of 7.6 ± 0.1 and 7.9 ± 0.1, respectively.

### Circular dichroism spectra of Bcp1

**Figure 10:**
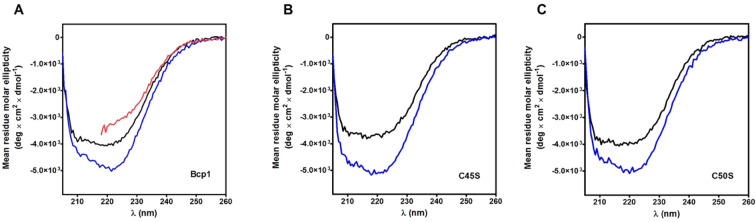
Circular dichroism spectra of Bcp1 and its mutants. (**A**) CD spectra of native Bcp1 (1.25 μM) (black line); rBcp1 (1.25 μM) in 0.2 M urea (blue line) and rBcp1 (1.25 µM) in 8 M urea (red line). The spectrum of rBcP1 in 8 M urea cannot be extended below 218 nm due to the interference of 8 M urea. (**B**) CD spectra of C45S (1.25 µM) (black line) and rC45S (1.25 μM) in 0.2 M urea (blue line). (**C**) CD spectra of C50S (1.25 µM) (black line) and rC50S (1.25 µM) in 0.2 M urea (blue line). CD spectra for the three proteins were recorded at pH 7.4, 25 °C.

The CD spectra of the native oxidized Bcp1 and of the reduced enzyme are shown in Fig. 10A. The reduced enzyme displays a well evident increased structuration due to some conformational change (Fig. 10A). Previous X-ray studies performed on the native and on the double cysteines mutant showed that the reduction caused an increase of α-helix (residues 43–54) instead of two small antiparallel β-strains^11^. Surprisingly, even the C45S and C50S mutants display a very similar trend, *i.e*. higher structuration after reduction (Fig. 10B,C). As both these mutants, expressed in *E. coli*^11^ do not display any titratable cysteines even in 8 M urea, they are probably present as mixed disulfide with GSH or other thiols coming from the host organism used to express this protein. This suggests that the formation of the protein mixed disulfide C45-C50 does not trigger the above reported structural changes but it probably follows a first glutathionylation event. A more accurate analysis of the CD spectra, performed as described in Materials and Methods section, reveals that reduced Bcp1 has the same α-helix content (33%) but 38% instead of 14% of β-sheet present in the native protein. For the two reduced mutants there is a similar or even higher increase of β-sheet (from 15% to 50%) and a decrease of 6% of the α-helix structure (from 40% to 34%).

### *Ss*PDO reactivity

**Figure 11:**
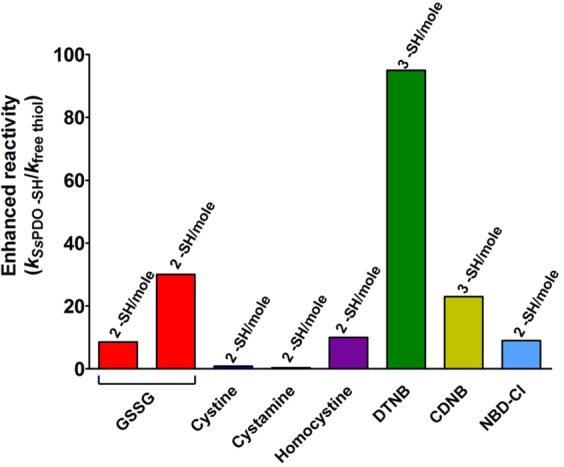
Reactivity of r*Ss*PDO cysteines. “Enhanced reactivity” of r*Ss*PDO toward disulfides and thiol reagents *i.e*. second order kinetic constants of r*Ss*PDO (*k*_*Ss*PDO -SH_) normalized to the constant calculated for an unperturbed protein cysteine for GSSG or normalized to the constants for free GSH for all other reagents (*k*_free thiol_) (see Table 3).

Native *Ss*PDO displays three disulfide bridges and no reduced cysteines^13^. Expression, purification and the reduced *Ss*PDO (r*Ss*PDO) were made as reported under Materials and Methods. Reaction with DTNB at pH 7.4 shows three hyper-reactive cysteines. The rate was too fast so this reaction was re-analyzed at pH 5.0. The second order kinetic constant was evaluated as 1900 M^−1^ s^−1^ that represents an incremental factor of about 100 when compared to an unperturbed protein cysteine (Table 3 and Fig. 11). DTNB is the only tested thiol reagent giving hyper-reactivity with this enzyme. Conversely, no or very small reactivity was found for GSSG and other natural disulfides.

**Table 3:** Reactivity of cysteines of fully reduced *Ss*PDO toward thiol reagents, pH 7.4.

Reagents	Free Cysteine	Free GSH	Reduced *Ss*PDO	
	*k* (M^−1^ s^−1^)	*k* (M^−1^ s^−1^)	*k* (M^−1^ s^−1^) (-SH/mole *Ss*PDO)	E.R.
GSSG	0.7^a^ (0.2)^b^	—	6 ± 1 (2)	8.57 30
Cystine	—	12	10 ± 2 (2)	0.83
Cystamine	—	55	20 ± 2 (2)	0.36
Homocystine	—	0.6	6 ± 1 (2)	10
DTNB^c^	—	20	1900 ± 110 (3)	95
CDNB	—	0.07	1.6 ± 0.1 (3)	23
NBD-Cl	—	8	72 ± 3 (2)	9

^a^Experimental value for free cys (p*K*_a_ = 8.55);

^b^Theoretical value for unperturbed protein cysteine (p*K*_a_ = 9.1);

^c^pH 5.0;

E.R., Enhanced reactivity;

Data are Mean ± S.D.

### p*K*_a_ determination of r*Ss*PDO

**Figure 12:**
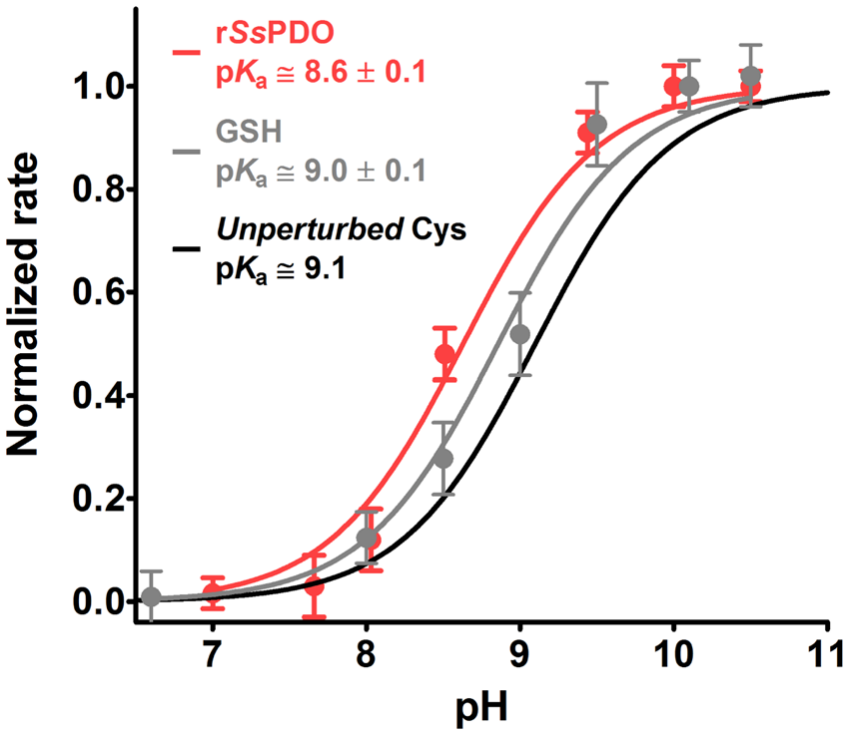
Average p*K*_a_ determination of *Ss*PDO. r*Ss*PDO (1 µM) was reacted with CDNB (1 mM) at variable pH values (red line). Average p*K*_a_ = 8.6 ± 0.1 of the three reactive cysteines in r*Ss*PDO. As a control experiment, GSH (100 µM) was reacted with CDNB (1 mM) at variable pH values (gray line). The error bars represent the S.D. from three independent experiments. The theoretical curve (black line) of an unperturbed protein cysteine (p*K*_a_ = 9.1) is also reported.

p*K*_a_ value was determined using CDNB. DTNB cannot be used as its reaction at high pH is too fast to be followed with a traditional spectrophotometer. Variation of the reaction rates of the protein cysteines at different pH values is shown in Fig. 12 and fulfilled an average p*K*_a_ of 8.6 ± 0.1, about 0.5 units lower than that of an unperturbed protein cysteine^18^. We underline that the average of p*K*_a_ of all cysteines coming from previous reported computational analysis is 9.33^13^.

### Circular dichroism spectra of *Ss*PDO

**Figure 13:**
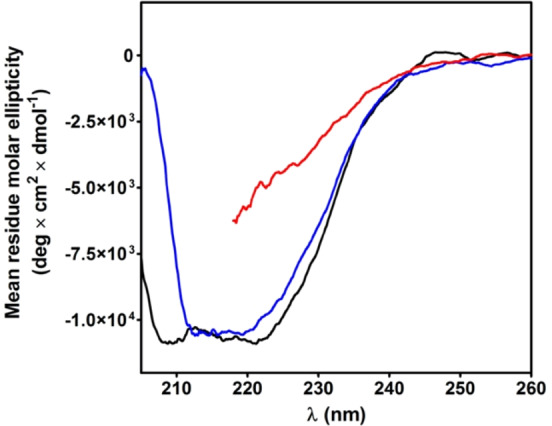
Circular dichroism spectra of *Ss*PDO and r*Ss*PDO. CD spectra of native *Ss*PDO (1 µM) (black line) in H_2_O, 25 °C; r*Ss*PDO (1 µM) in 0.6 M urea (blue line) and r*Ss*PDO (1 µM) in 8 M urea (red line) at pH 7.4, 25 °C. The spectrum of rSsPDO in 8 M urea cannot be extended below 218 nm due to the interference of 8 M urea.

The CD spectra of the native and reduced *Ss*PDO are reported in Fig. 13. Analysis with the on-line program BeStSel discloses that the native *Ss*PDO has 30% α-helix and 19% β-sheet while the reduced form has only 5% of α-helix and 47% of β-sheet.

## Discussion

This study discovers the presence of a single hyper-reactive cysteine toward GSSG in the reduced chymotrypsinogen. This hyper-reactivity fulfills an incremental kinetic factor of 1375, a value similar to that found in albumin (> 1250 for Cys75)^1^, in lysozyme (3000 for Cys94)^2^ and in ribonuclease (3500 for Cys95)^3^. This phenomenon represents a novelty for all these proteins. Many studies, in fact, were performed in the past to detail the oxidative pathway of these enzymes^20–24^. However, no data have been reported about the intrinsic reactivity of specific cysteines toward natural disulfides as well as toward thiol reagents. The formation of correct disulfide bridges is generally attributed to the action of the protein disulfide isomerase (PDO) and ER oxidoreductin 1 (Ero1) enzymes without any active participation of specific protein cysteines. The possible action of GSSG in this process is also poorly considered despite its high concentration in the endoplasmic reticulum where oxidative folding occurs. The discovery that in several proteins, reduced to the state of molten globules, specific cysteines show extraordinary reactivity towards GSSG suggests that this could represent the real ‘incipit’ of their oxidative folding. In the case of chymotrypsinogen, mass spectrometry experiments identified Cys1 as the hyper-reactive residue toward GSSG (see Fig. 3). As demonstrated for albumin, lysozyme and ribonuclease, even in the case of chymotrypsinogen, the hyper-reactivity toward GSSG cannot be explained only on the basis of a lowered p*K*_a_. In fact, the p*K*_a_ of 8.1, about 1 unit lower than an unperturbed cysteine, may cause only about tenfold higher reactivity toward GSSG^2^. The crucial factor was identified as a transient rChTg-GSSG complex (*K*_D_ = 1.5 mM) indicated by the fluorescence experiments (Fig. 5). Again the reduced protein, even if assembled in oligomeric or polymeric structures, shows a sophisticated kinetic property toward GSSG which may be important during the oxidative process. In fact, a very fast glutathionylation of a single cysteine could represent the “incipit” of the oxidative folding, whose utility can be only speculated. In fact, in the case of lysozyme, the rapid glutathionylation of Cys94 stops completely the deleterious aggregation at pH 7.4^2^, while the glutathionylation of Cys1 in rChTg at this pH could not be detected due to a too fast protein aggregation (data not shown). However, assuming an enhanced reactivity at pH 7.4 similar to that observed at pH 5.0, we can speculate that the glutathionylation of Cys1 may occur within few seconds during the nascent phase, where the aggregation is probably negligible due to a “very low” enzyme concentration.

Of particular interest is the lack or very low hyper-reactivity toward GSSG in two proteins that are produced in an organism where GSH and GSSG are not present^9,12^. In fact, both rBcp1 and r*Ss*PDO show only 2 and 30 times higher reactivity toward GSSG, respectively. The estimated p*K*_a_ of cysteines in these two proteins allows interesting conclusions. We noted that the p*K*_a_ of Cys1 in rChTg is 8.1 while both Cys50 and Cys45 show a lower p*K*_a_ of 7.6 and 7.9, but they are almost unreactive toward GSSG. Thus, the extraordinary hyper-reactivity of Cys1 in rChTg must be mainly attributed to a productive reversible protein-GSSG complex, as previously proposed for lysozyme and ribonuclease^2,3^. The similar p*K*_a_ values found for Cys45 (in rC50S) and Cys50 (in rC45S) in Bcp1 suggest that these two residues may have a similar reactivity for DTNB, but once modified one residue, the other becomes non accessible to this reagent. This idea is confirmed by the unique titratable cysteine with DTNB in the native enzyme as well as in both mutants. The simple scheme reported in Fig. 7B summarized this evidence.

In conclusion, what described in this study seems to exclude that the cysteine hyper-reactivity toward GSSG found in chymotrypsinogen is a casual event. On the contrary, the finding that other disulfide containing proteins like albumin, lysozyme, ribonuclease and now chymotrypsinogen shows a specific super-reactive cysteine when taken to their molten globule-like conformations, may be a signal for the existence a precise evolutionary purpose for many proteins.

What does it happen in the absence of this hyper-reactivity? A direct mutation of the hyper-reactive cysteine will not be a good experimental choice to obtain useful response, as it will alter deeply the disulfide scenario. A convincing answer could come from suitable site mutations in these proteins causing a decreased affinity for GSSG. Obviously, such target will be object of future investigations.

A further possible role of the ultra-rapid glutathionylation of a single cysteine is to limit the formation of non-natural disulfides, which may be more frequent in a disordered structure. In the ancestral microorganisms the lack of GSH/GSSG and the low reactivity of the protein cysteines versus this dithiol open a new debate on evolution mechanism of the oxidative folding.

A good indication that a fast reaction of Cys1 with GSSG may avoid incorrect oxidative folding comes from the observation that lowering the level of glutathione within the mammalian cell leads to the formation of non-native disulfide bonds^25^.

We believe that the discovery of this phenomenon is a completely new piece that must be inserted in the complex “puzzle” of the oxidative folding of proteins^26^ and that may be important to clarify the origin of misfolded diseases^27^ like Alzheimer or Parkinson.

From a more general perspective, the discovery that a protein cysteine reactivity can be enhanced by other factors, besides that of the lowering of its p*K*_a_, is an interesting finding. Cysteine is a key residue involved in many catalytic mechanisms or in crucial protein functions and its modification may regulate many enzymes and inhibit a lot of functional proteins. Thus, the design and synthesis of drugs able to interact specifically and rapidly with specific cysteines is an emerging and attractive target for pharmacologists and biochemists. It is generally accepted that cysteine reactivity is mainly driven by its p*K*_a_, given that only the thiolate form is active in all its reactions. Our results indicate that two different factors may have much more influence i.e. a selective and specific binding which may favor the interaction of a single residue with a given reagent (in our case GSSG) and a more nonspecific interaction with thiol reagents with hydrophobic character (in our case CDNB, DTNB and NBD-Cl). As previously demonstrated a p*K*_a_ variation cannot increase the intrinsic reactivity of a cysteine more than 40–50 times^2^. The discovery that these two different factors may enhance it up to hundred and even thousand times may be important and useful for the above mentioned research areas.

## Materials and Methods

### Chemicals and reagents

α-Chymotrypsinogen A (ChTg) from bovine pancreas. The protein was tested for purity by using an FPLC system (Amersham-Pharmacia, Sweden) equipped with a column Superose-12 (data not shown). Bromopyruvic acid (BrP), cystamine, L-cysteine (Cys), 1-chloro-2,4-dinitrobenzene (CDNB), 5,5′-dithiobis(2-nitrobenzoic acid) (DTNB), dithiothreitol (DTT), ethylendiamminotetreaacetic acid (EDTA), L-glutathione (GSH), oxidized glutathione (GSSG), homocystine, N-acetyl-L-tryptophanamide (NATA), 4-chloro-7-nitrobenzofurazane (NBD-Cl), and all other reagents were from Sigma-Aldrich (St. Louis, Mo, USA). GSH solutions were freshly prepared and the amount of GSSG was less than 1% as assayed by standard analytical procedures.

### Proteins expression and purification

Bcp1 and its mutants, C45S and C50S, from *S. solfataricus* were expressed and purified in *E. coli* as previously reported^10,11^. Briefly, *E. coli* transformed strains: BL21(DE3)-RIL/pET30Bcp1, BL21(DE3)-RIL/pETC45S, BL21(DE3)-RIL/pETC50S were grown in selective medium and the expression of recombinant proteins were induced by IPTG. The cells were harvested by centrifugation and disrupted by sonication. The soluble fractions were heat-treated at 80 °C for 15 min. The recombinant proteins were purified at the homogeneity by affinity chromatography on HisTrap HP (GE Healthcare) and analyzed by SDS-PAGE revealing a single band of 18 kDa for each protein.

Recombinant *Ss*PDO was expressed in *E. coli* and purified to homogeneity, as previously reported^12^ by a three-step purification procedure consisting of a thermal precipitation step at 80 °C for 20 min, an affinity chromatography on a HisTrap HP (GE Healthcare) followed by an anionic chromatography on a Resource Q (GE Healthcare). SDS/PAGE of the final preparation revealed a single band with a molecular mass of about 27 kDa.

### Proteins reduction

The ChTg concentration was estimated in 1 mM HCl at 280 nm (ε = 50585 M^−1^ cm^−1^)^28^. The ChTg reduction was performed by solubilizing the protein (8 mg), without further purification, in 8 M urea, 1 mM EDTA, 10 mM sodium borate buffer pH 8.5 (1 mL) and by adding 5.2 mM DTT (ChTg:DTT = 1:20). After 50 minutes at 60 °C, the reduced protein was purified from DTT excess by passing through a Sephadex G-25 column (1 × 20 cm) equilibrated with 8 M urea, 1 mM EDTA, 10 mM acetate buffer pH 5.0. Through the manuscript, the reduced chymotrypsinogen after this process is indicated as rChTg. The titration of the protein cysteines was performed at pH 5.0 with 1 μM rChTg, 0.05 mM DTNB in the presence of 0.2 M urea by following the release of TNBS^-^ (ε_Μ_ = 11800 M^−1^cm^−1^ at 412 nm, pH 5.0)^2^. The reaction was complete within five minutes. A similar titration experiment in 8 M urea at pH 8.0 needed 10 min of reaction (ε_Μ_ of TNBS^-^ = 14100 M^−1^cm^−1^ at 412 nm, pH 8.0)^2^. The reduced enzyme was freshly prepared every day and its -SH content at pH 5.0 and 0.2 M urea (25 °C) remained constant throughout the experimental session.

The concentration of Bcp1 and the two mutants C45S and C50S was evaluated in water at 280 nm considering an extinction coefficient of 16055 and 15930 M^−1^ cm^−1^, respectively^28^. Bcp1 (0.125 mM), C45S (0.154 mM) and C50S (0.141 mM) were individually denatured in 8 M urea, 10 mM sodium borate buffer pH 8.5 in the presence of 1 mM EDTA and reduced with DTT (Protein:DTT = 1:10) at 60 °C. After 30 minutes, each protein solution was loaded on a Sephadex G-25 column (1 × 20 cm) equilibrated with 2 M urea, 1 mM EDTA, 10 mM phosphate buffer pH 7.4. The abbreviations for reduced proteins are rBcp1, rC45S, and rC50S in the manuscript. The total number of protein cysteines (2 -SH in rBcp1 and 1 -SH in the two reduced mutants) was measured with DTNB at pH 7.4 only in the presence of 10 M urea, while 1 -SH (rBcp1), 0.6 -SH (rC45S) and 0.65 -SH (rC50S) were titrated in the presence of 0.2 M urea.

The *Ss*PDO concentration was measured in water at 280 nm (ε = 20775 M^−1^ cm^−1^)^28^. The denaturation and the reduction of *Ss*PDO was executed in 8 M urea, 1 mM EDTA, 10 mM sodium borate buffer pH 8.5 in the presence of DTT (*Ss*PDO:DTT = 1:6) at 40 °C. The reduced protein is abbreviated in r*Ss*PDO. After 20 minutes, -SH were titrated with DTNB at pH 5.0 in 0.4 M urea.

### Reactivity of rChTg toward GSSG, disulfides and thiol reagents

The reactions of rChTg towards GSSG, cystine, homocystine, and cystamine were assayed indirectly incubating 5 µM of reduced protein with 1 mM of each disulfide in a total volume of 6 ml of 20 mM sodium acetate buffer pH 5.0, 0.2 M urea (25 °C) except 10 mM acetate buffer for GSSG. At different incubation time an aliquot of the reaction mixture was treated with HCl 14 mM (final concentration) to reach an acidic condition around pH 2.0, then 0.55 ml of this solution was centrifuged at 14000 × g 10 min on Vivaspin-500 (10 KDa membrane cut-off) (GE Healthcare, UK). The filtrate was brought to pH 8 with 1 M phosphate buffer and thiols contents were titrated with DTNB (ε_TNBS_^-^ = 14100 M^−1^ cm^−1^ at pH 8.0)^2^. The incubation times were very different for each disulfide: until around 67 hours for GSSG and homocystine, 25 hours for cystamine, and around 7 hours for cystine.

The reactivity of sulfhydryl groups of rChTg toward CDNB, NBD-Cl, and DTNB was estimated as described follow. The reactivity toward CDNB was evaluated spectrophotometrically in continuous at 340 nm where the Cys-DNB adduct absorbs (ε = 9600 M^−1^ cm^−1^)^1^. rChTg (5 µM) was reacted with 0.5 mM CDNB in 50 mM sodium acetate buffer pH 5.0, both in 0.28 M and 8 M urea (25 °C). The reaction of rChTg (5 µM) toward NBD-Cl (50 µM) was determined spectrophotometrically at 419 nm were the Cys-NBD adduct absorbs (ε = 13000 M^−1^ cm^−1^)^29^ in 50 mM sodium acetate buffer pH 5.0, 0.28 M urea (25 °C). The reactions of rChTg with DTNB were performed using a SFA-12 Rapid Kinetics Accessory (Hi-Tech Scientific, Bradford-on-Avon, UK). The reactivity of rChTg (0.6 µM) toward DTNB (50 µM) was evaluated at 412 nm where TNBS^-^ absorbs (ε_TNBS_^-^ = 11800 M^−1^ cm^−1^ at pH 5.0)^2^ in 50 mM sodium acetate buffer pH 5.0, 0.2 M urea (25 °C); the same conditions but in spectrophotometric continuous acquisition without rapid-mixing modality were applied for the reaction in 8 M urea. Only to calculate the kinetic constant for the single hyper-reactive cysteine the experimental conditions were rChTg at 0.23 µM, DTNB 10 µM in 50 mM sodium acetate buffer pH 5.0, 0.2 M urea (25 °C).

Reactivity of free cysteine with GSSG and free GSH with other reagents were evaluated as reported in our previous studies^1–3^ with slightly modified protocol increasing the acquisition-time to 2 hours due to the pH 5.0.

### Reactivity of rBcp1, rC45S, rC50S toward disulfides and other thiol reagents

The reactivity of sulfhydryl groups of rBcp1 toward disulfides was assayed in the following condition: protein 1.25 µM final concentration, in 10 mM potassium phosphate buffer pH 7.4, 0.2 M urea (25 °C), the concentrations of each disulfide were 1 mM for GSSG, cystine, cystamine, and 0.5 mM for homocystine. After different incubation times (from 30 seconds to 10 minutes) the reactions were stopped with sodium acetate buffer 1 M pH 5.0 and then the disappearance of the reactive cysteines of rBcp1 was determined using DTNB (50 µM) as titrant (25 °C). The reactivity toward DTNB (50 µM) was followed spectrophotometrically at 412 nm where TNBS^-^ absorbs (ε_TNBS_^-^ = 11800 M^−1^ cm^−1^ at pH 5.0) in 0.1 M sodium acetate buffer pH 5.0, 0.12 M urea (25 °C). While the reactivity toward CDNB (1 mM) and NBD-Cl (0.1 mM) was performed in a potassium phosphate buffer 0.1 M pH 7.4, 0.12 M urea (25 °C) at the wavelength of 340 and 419 nm where Cys-DNB adduct (ε = 9600 M^−1^ cm^−1^) and Cys-NBD adduct (ε = 13000 M^−1^ cm^−1^) absorb.

The experimental conditions for the reactivity were substantially the same for the two mutants; rC45S and rC50S (3.8 µM) were incubated with 1 mM cystine and cystamine (for 1 minute) and with 0.5 mM homocystine (for 5 minutes) in potassium phosphate buffer 0.1 M pH 7.4, 0.2 M urea (25 °C). The amount of cysteine or cysteamine released was determined by adding 1 mM BrP forming cyclic sulfur products (lanthionine ketimine and aminoethylcysteine ketimine) that absorb at 296 nm (ε = 6200 M^−1^ cm^−1^), while after adding NaOH (20 mM final concentration) to the solution the homocysteine released was determined by the reaction with 1 mM BrP. The corresponding cyclic ketimine sulfur product (cystathionine ketimine) absorbs at 296 nm (ε = 3200 M^−1^ cm^−1^)^30^. The reactions of rC45S and rC50S with DTNB were performed using rapid-mixing apparatus. The reactivity of rC45S (3.8 µM) and rC50S (2.6 µM) toward DTNB (50 µM) was evaluated at 412 nm where TNBS^-^ absorbs (ε_TNBS_^-^ = 14100 M^−1^ cm^−1^ at pH 7.4 for rC45S; ε_TNBS_^-^ = 12400 M^−1^ cm^−1^ at pH 6.0 for rC50S)^1^ in potassium phosphate buffer 0.1 M, 0.2 M urea (25 °C). The choice of pH derived from the different solubility of the mutants. Finally, the reactivity of rC45S (3.8 µM) and rC50S (2.6 µM) toward CDNB (1 mM) and NBD-Cl (0.1 mM) was performed in a potassium phosphate buffer 0.1 M pH 7.4, 0.2 M urea (25 °C) at the wavelength of 340 and 419 nm where Cys-DNB adduct (ε = 9600 M^−1^ cm^−1^) and Cys-NBD adduct (ε = 13000 M^−1^ cm^−1^) absorb. Reactivity of free cysteine with GSSG and free GSH with other reagents were evaluated as reported in our previous studies^1–3^. The second order kinetic constant between GSH and DTNB at pH 7.4 (with slightly modified protocol) was obtained using the rapid-mixing apparatus.

### Reactivity of r*Ss*PDO toward disulfides and thiol reagents

The reactions of r*Ss*PDO (0.9 µM final concentration) with GSSG, cystine, cystamine, and homocystine (all disulfides at 1 mM final concentration) were measured by incubating the reagents in 10 mM potassium phosphate buffer pH 7.4, 0.4 M urea (25 °C). After 1 minute the reactions were stopped with acetate buffer 1 M pH 5.0 and then the disappearance of the reactive cysteines of r*Ss*PDO was determined using DTNB (20 µM) as titrant (25 °C). The reactions among r*Ss*PDO and DTNB (20 µM), CDNB (1 mM), NBD-Cl (0.1 mM) were performed in sodium acetate buffer 0.1 M pH 5.0, 0.4 M urea (25 °C) for DTNB and potassium phosphate buffer 0.1 M pH 7.4, 0.4 M urea (25 °C) for CDNB and NBD-Cl. The wavelengths for the spectrophotometric assays and the relative extinction coefficients were 412 nm (ε_TNBS_^-^ = 11800 M^−1^ cm^−1^ at pH 5.0), 340 nm (ε_Cys-DNB_ = 9600 M^−1^ cm^−1^ at pH 7.4) and 419 nm (ε_Cys-NBD_ = 13000 M^−1^ cm^−1^ at pH 7.4) for DTNB, CDNB and NBD-Cl respectively. Reactivity of free cysteine with GSSG and free GSH with other reagents were evaluated as reported in our previous studies^1–3^.

### p*K*_a_ determination

The determination of the average p*K*_a_ of available cysteines of rChTg (0.6 μM) was measured with CDNB (1 mM) in a solution of 0.1 M Britton-Robinson buffers (pH varying from 6.0 to 10.0) at 25 °C using the rapid-mixing apparatus. The reaction rate between rChTg and CDNB was subtracted by the aggregation rate of rChTg at each experimental pH examined. The average p*K*_a_ of rBcp1 (1.25 μM), rC45S (2.6 μM) and rC50S (2.6 μM) was determined with DTNB (20 μM) in a solution of 20 mM Britton-Robinson buffers (pH varying from 5.0 to 10.0), 0.2 M urea at 25 °C using the rapid-mixing apparatus. Appropriate extinction coefficients at 412 nm for TNBS^-^ were considered below pH 7.0. Finally, the average p*K*_a_ of 1 μM r*Ss*PDO with 1 mM CDNB was estimated in a solution of 20 mM Britton-Robinson buffers (pH varying from 7.0 to 10.0), 0.4 M urea at 25 °C. The data for all the protein studied are displayed as normalized rates calculated from observed initial velocities divided by the maximum velocity measured at full deprotonation. All p*K*_a_ values were calculated by a curve fitting analysis.

### Circular dichroism spectroscopy

The CD spectra of ChTg and rChTg (1.3 μM final concentration) were measured in 10 mM sodium acetate buffer pH 5.0 at 25 °C, for rChTg the buffer contain 0.2 M or 8 M urea. The CD spectra of *Ss*PDO (1.0 μM final concentration) were obtained in deionized water, while the spectra of r*Ss*PDO (1.0 μM final concentration) were determined in 10 mM potassium phosphate buffer pH 7.4 at 25 °C, the buffer contain 0.2 M or 8 M urea. The setting panel for ChTg and *Ss*PDO of the spectropolarimeter Jasco J-600 (Easton, MD) was: slit 2 nm, sensibility 20 mdeg, range 205–260 nm, resolution 0.2 nm; using a quartz cuvette of 0.5-cm path length. The CD spectra of Bcp1 and rBcp1 (1.25 uM final concentration) were obtained in 10 mM potassium phosphate pH 7.4 at 25 °C, for rBcp1 the buffer contain 0.2 M or 8 M urea. The CD spectra of Bcp1 mutants were acquired in the same condition except for the 8 M urea. The setting panel of the spectropolarimeter Jasco J-1500 (Easton, MD) was: slit 2 nm, sensibility 20 mdeg, range 205–260 nm, resolution 0.5 nm; using a quartz cuvette of 0.5-cm path length.

The analysis of circular dichroism spectra were performed using BeStSel server^31^.

### Effect of urea on the reactivity of rChTg

The effect of urea concentration on the reactivity of rChTg cysteines was assayed using DTNB as follows: 0.6 μM rChTg was incubated with 47.5 μM DTNB in 50 mM sodium acetate buffer, pH 5.0 (25 °C) containing variable concentrations of urea (from 0.2 M to 8 M). The reaction rate was measured spectrophotometrically at 412 nm using the rapid-mixing apparatus. The reaction of free cysteine or GSH (10 μM) with DTNB (50 μM) at different urea concentrations (pH 5.0, 25 °C) was used as a control to verify possible effects of urea on the reaction rate.

### Fluorescence analysis of rChTg

The fluorescence measurements were performed on a Fluoromax-4 Horiba spectrofluorometer with slits 1–4 nm, excitation wavelength 280 nm, emission spectra 320–380 nm, temperature 25 °C, with a quartz cuvette of 1-cm path length. The spectra of 0.5 µM rChTg in 0.2 M urea, 10 mM sodium acetate buffer pH 5.0 were recorded in the presence of different GSSG concentrations (from 0 to 5 mM). The maximum intensities of each spectra were subtracted by the intensities acquired for NATA in the same conditions.

### Identification of the hyper-reactive cysteine of rChTg by mass-spectrometry

rChTg (5 μM) was incubated with GSSG (1 mM) in 10 mM sodium acetate buffer, pH 5.0 in the presence of 0.3 M urea at 25 °C. After 20 minutes, the reaction was stopped by adding 10 mM BrP that alkylates residual protein cysteines within 1–2 sec. Then the sample was lyophilized. A reduced ChTg (5 μM) solution was immediately alkylated with BrP (10 mM) in 0.3 M urea, 10 mM acetate buffer pH 5.0 and used as control.

Mass spectrometry analysis was performed basically in the same manner of our previous study^3^. The procedures are described as follow. Samples were resuspended in 0.1% trifluoroacetic acid (TFA) and desalted by reversed-phase HPLC on a Phenomenex Jupiter C4 column (250 mm × 2.0 mm, 300 Å pore size) with a linear gradient from 10% to 95% of solvent B (0.07% TFA in 95% acetonitrile) in 30 min, at a flow rate of 200 μL/min using an Agilent Technologies 1100 HPLC (Agilent Technologies, USA)^3^. Protein fractions were collected and lyophilized. Pepsin hydrolysis was carried out by dissolving the samples in 5% formic acid, pH 2.5 and adding pepsin at an enzyme to substrate ratio of 1:50 w/w at 37 °C for 2 hours. Samples were then lyophilized and resuspended in 0.2% formic acid and the samples were then directly analyzed by nanoLC/MS-MS on an Orbitrap Elite mass spectrometer equipped with a nanoHPLC (ThermoFisher, USA)^3^. Peptides containing modified cysteine residues were selected using the ion extraction chromatograms of the corresponding multiply charged ions and the assignments were confirmed by manual inspection of their fragmentation spectra.

### Data and graphical representation

When necessary the experimental data reported in Figures and Tables were analyzed and expressed as Mean ± Standard Deviation (S.D.). Generally, through the manuscript, data were obtained from independent experiments (from three to ten) performed in different days by the same operators using the same instruments. The graphic and results visualization were obtained using GraphPad Prism software v5.0 (La Jolla, CA, USA). Crystal structure of chymotrypsinogen is derived by PDB id: 2CGA^32^, the structure of Bacterioferritin Comigratory Protein 1 is derived by PDB id: 3DRN^11^ and elaborated by WinCoot-0.8.9^33^. Finally, three-dimensional structures were drawn by UCSF chimera^34^.
